# The synergistic effect of EMT regulators and m6A modification on prognosis-related immunological signatures for ovarian cancer

**DOI:** 10.1038/s41598-023-41554-y

**Published:** 2023-09-08

**Authors:** Yanna Zhang, Xun Wang, Xiaogang Duan, Ting Du, Xiancheng Chen

**Affiliations:** 1 Department of Blood Transfusion, Sichuan Provincial People’s Hospital, University of Electronic Science and Technology of China, Chengdu, 610072 People’s Republic of China; 2 Department of Biotherapy, Cancer Center and State Key Laboratory of Biotherapy, West China Hospital, Sichuan University, Chengdu, Sichuan 610041 People’s Republic of China; 3 Chengdu Eighth People’s Hospital/Geriatric Hospital of Chengdu Medical College, Chengdu, 610000 Sichuan People’s Republic of China; 4 Noncoding RNA and Drug Discovery Key Laboratory of Sichuan Province, Chengdu Medical College, Chengdu, 610000 Sichuan People’s Republic of China

**Keywords:** Cancer, Biomarkers

## Abstract

Recently, there has been growing interest among researchers in exploring the effects of epithelial-mesenchymal transformation (EMT) or N6-Methyladenosine (m6A) modification regulators on tumor development. However, the synergistic efficiency of these regulators in relation to ovarian cancer development remains unclear. This study aims to explore the transcription patterns of main regulators, including 19 EMT and 22 m6A, in ovarian cancer samples from TCGA datasets and normal samples from GTEx datasets. After conducting a LASSO regression analysis, ten prognostic signatures were identified, namely KIAA1429, WTAP, SNAI1, AXL, IGF2BP1, ELAVL1, CBLL1, CDH2, NANOG and ALKBH5. These signatures were found to have a comprehensive effect on immune infiltrating signatures and the final prognostic outcome. Next, utilizing the ssGSEA algorithm and conducting overall survival analyses, we have identified the key prognosis-related immunological signatures in ovarian cancer to be ALKBH5, WTAP, ELAVL1, and CDH2 as the regulators. The characteristic immune response and related genetic expression have revealed a significant correlation between the alteration of m6A regulators and EMT regulators, indicating a synergistic effect between these two factors in the development of ovarian cancer. In summary, our research offers a novel perspective and strategy to enhance the occurrence, progression, and prognosis of ovarian cancer.

## Introduction

So far, ovarian cancer remains the most lethal form of female malignancy worldwide, regardless of the diverse histologic subtypes^1,2^. As there are typically minimal or no noticeable symptoms during the early stages, diagnosis of this condition is often delayed until the advanced stage, earning it the nickname of the “silent killer”^3,4^. Although the treatment for ovarian malignancy is constantly evolving, the 5-year survival rates still remain below 45%^5^. As a result, there exist significant challenges for implementing novel strategies aimed at enhancing the survival rate and quality of life for those suffering from ovarian cancer. According to some investigations, both epithelial-mesenchymal transition (EMT) and N6-Methyladenosine (m6A) modification play a role in the development and occurrence of several tumors^6–10^. EMT refers to a complex and reversible process involving the loss of epithelial apical-basal polarity and cell junctions, and the acquisition of migratory capabilities under specific conditions. This process plays a critical role in the progression of tumor occurrence, development, invasion, metastasis, and drug resistance^11,12^. Importantly, the EMT process plays a critical role in the invasion and metastasis of ovarian cancer^13,14^. An RNA methylation modification called m6A occurs at the nitrogen-6 position of the adenosine base^14,15^. Simultaneously, m6A modification, an integral part of the epigenetic pattern, is a commonly occurring and classic co-transcriptional alteration in eukaryotes, which has garnered significant interest among researchers in recent years^14,16^. Previous research has established that m6A methylation plays a central role in several physiological and pathological processes, especially during the onset and progression of various types of cancers^17,18^. Methylation modification of m6A, which is similar to DNA and protein modification, is dynamically regulated by Writers (methyltransferases), Erasers (demethylases), and Readers (reading proteins)^19,20^. Many studies have demonstrated that m6A modification can impact tumor formation and evolution by regulating biological functions associated with cancer^21,22^. Although the accumulating data suggests that both EMT and m6A may play vital roles in the administration of certain physiological or pathological processes^22–24^, our current understanding of their effects on the development and prognosis of ovarian cancer remains incomplete. So far, the relevant mechanisms underlying the prognosis and tumor immune microenvironment in ovarian cancer remain insufficient and ambiguous. Additionally, the tumor heterogeneity further complicates the critical evaluation of each patient's prognosis. Accurately evaluating the prognosis and improving the survival rate of ovarian cancer patients continue to be significant challenges.

While the aforementioned research often focuses solely on EMT or m6A regulators^25,26^, it should be noted that the impact on tumors is typically the result of a highly synergistic process involving a variety of tumor suppressors. Thus, gaining an integrated understanding of the synergistic effects of multiple EMT and m6A regulators on the prognostic and immune characteristics would aid in comprehending their crucial roles in the progression of ovarian cancer. In our study, we conducted a systematic analysis of the expression patterns of EMT and m6A regulators in 379 ovarian cancer samples from TCGA datasets and 88 normal ovarian samples from GTEx datasets. Our aim was to gain a comprehensive understanding of the potential relationship between these patterns, immune characteristics, and prognosis outcomes. LASSO regression and ssGSEA analysis have revealed that EMT and m6A regulators play a critical role in the immune microenvironment and prognosis of ovarian cancer. Next, we combined survival analysis with immune response and characteristic alterations to identify core prognosis-related immunological signatures. This allowed us to further validate their impact on the immune microenvironment and prognosis estimation for ovarian cancer (Figure S1). In order to offer a fresh approach in developing more efficient strategies for enhancing the development and prognosis of ovarian cancer.

## Materials and methods

### Collection of relevant sample data

The transcriptome profiles and relevant clinical information of patients with ovarian cancer were derived from TCGA (https://portal.gdc.cancer.gov/) and normal human ovarian samples were obtained from GTEx (https://www.gtexportal.org/home/datasets). Then these data were combined with batch normalization using the R package “sva”.

### Screening and transcriptional characterization of EMT and m6A modification regulators

A list of 19 EMT regulators and 22 m6A modification regulators were collected from published literatures^20,27–32^. Next, the transcriptional expression characterization between the ovarian cancer and normal samples were systematically contrasted by R software (version 3.6.3).

### Construction and analyzing the PPIs network

The Protein–protein interactions between EMT and m6A regulators were explored by STRING (version 11.5, https://www.string-db.org/)^33^. In order to obtain a more credible PPIs network, the regulators with interaction score greater than 0.4 were only obtained and exhibited by Cytoscape (Version 3.6.1)^34^.

### Correlation between EMT and m6A regulators

The co-modulation regulators were identified according to the PPIs among EMT and m6A regulators. Meanwhile, the expression correlation between EMT and m6A regulators was calculated by the package “ggpubr” and “ggExtra” based on “ggplot2” in R software (version 3.6.3).

### Acquisition of prognostic characteristics based on EMT and m6A regulators

The prognostic scores for EMT and m6A regulators in ovarian cancer were evaluated through univariate independent prognostic analysis. Combined the prognostic regulators with co-modulation regulators, the risk characteristics were explored via LASSO algorithm^35^. Then, prognostic signatures and their weight coefficients were calculated by the minimal loss as the optimal norm factor λ related to the ovarian cancer samples. The risk score of samples was evaluated via function: ∑Weight*xi, where Weight is the coefficient factor, xi is the expression value of z-score conversion for the regulator. The formula was used to verify the risk score of every ovarian cancer sample. Then, ovarian cancer samples were divided into high risk and low risk groups through the median risk scores. ROC analysis was accomplished based on the risk score to define whether the survival prediction was sensitive and specific.

### Assessment of immunocyte and tumor microenvironment infiltration for prognostic characteristics

According to EPIC (https://github.com/GfellerLab/EPIC), there are eight immunocyte types consisted of B cells, CD4 + T cells, CD8 + T cells, NK cells, Cancer associated fibroblast cells, endothelial cells, macrophages and uncharacterized cells to evaluate the effect and correlation of immunocyte infiltration on the prognostic characteristics. Meanwhile, tumor purity, stromal cell score, adipocytes and microenvironment score were performed using R package “ESTIMATE” or xCell (version 1.0). In addition, these infiltration levels in high risk or low risk subtype were also further investigated.

### Confirmation the accuracy for immune clustering

According to the expression patterns of ovarian cancer samples, the Tumor Purity, ESTIMATE Score, Immune Score, and Stromal Score were identified via using R package “ESTIMATE”^36^ and validated the effectuality of ssGSEA grouping^37^. The score deriving from the results of “ESTIMATE” was visualized by heatmap and violin plot. Next, combined with clinical information on ovarian cancer samples, survival analysis was performed among three clusters through using package “survival”. In addition, we performed the R package “CIBERSORT” to evaluate the differences of 22 immune cell subtypes among three clusters on the foundation of ovarian cancer expression file^38^. Finally, the expression patterns of prognostic characteristic regulators in three clusters were analyzed via the package “ggpubr”.

### Prediction for immune response

Spearman correlations between the expression or methylation of prognosis-related immunological characteristic regulators and Immunomodulator (including Immunoinhibitors, Immunostimulator, and MHC molecules) and associations between expression and molecular subtypes (covering Differentiated, Immunoreactive, Mesenchymal and Proliferative) across ovary cancer were calculated using TISIDB database^39^ (http://cis.hku.hk/TISIDB/).

### Immunotherapeutic response prediction

The tumor immune dysfunction and exclusion (TIDE) algorithm (http://tide.dfci.harvard.edu/) was used for predicting the clinical response to immune checkpoint block therapy using the transcriptomic profile of ovarian cancer. Moreover, the unsupervised subclass mapping (https://cloud.genepattern.org/gp/pages/login.jsf) method was further applied to predict the responsiveness to immune checkpoint block therapy of different risk or immune subtypes.

### Genetic alteration of prognosis-related immunological signatures in ovarian cancer

CBioPortal^40,41^(version 5.3.12, http://www.cbioportal.org/) was used to analyze and visualized the genetic alteration in key prognosis-related immunological signatures regulators in ovarian cancer.

### Key prognosis-related immunological regulators validation

Protein expression analysis of key prognosis-related immunological regulators was using data from CPTAC dataset in UALCAN^42,43^. Simultaneously, the expression about key prognosis-related immunological signatures regulators in various ovarian cancer cell lines were also explored from Cancer Cell Line Encyclopedia (CCLE)^44^.

### Statistical analyses

All the above analyses were performed using R 3.6.3 software and *P* < 0.05 was deemed to statistical significance.

### Ethics approval and consent to participate

All data are from public databases and do not involve ethical approval or consent to participate. And our manuscript was also not involved the subject.

## Results

### Transcriptional characterization of EMT and m6A regulators in ovarian cancer

**Figure 1:**
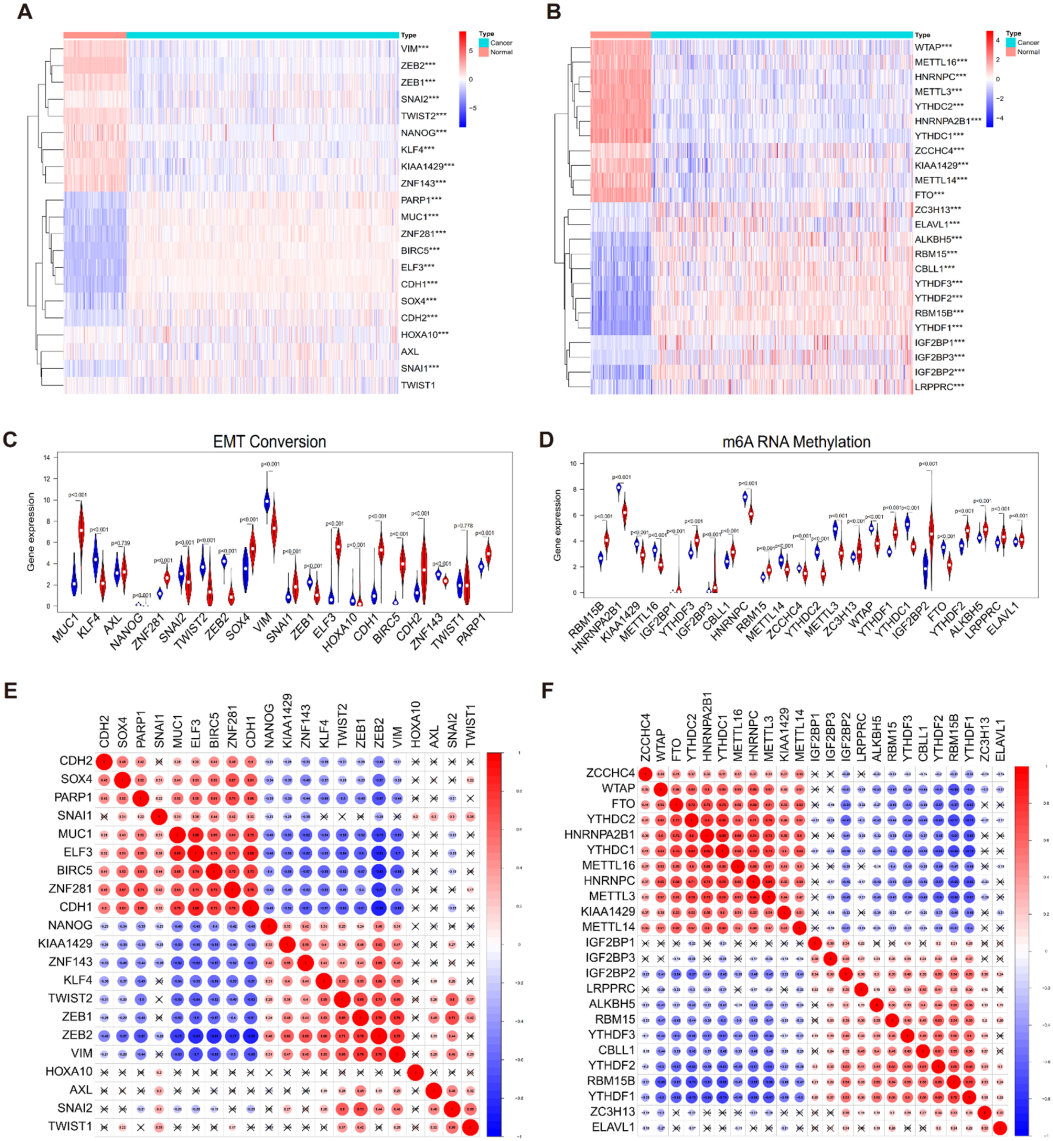
Expression landscape and interaction among EMT and m6A modification in ovary cancer. The expression levels of m6A regulators (**A**) and EMT regulators (**B**) in 88 normal samples and 379 ovary cancer samples were visualized via the package “pheatmap” in R software (Version 3.6.3). Quantitative analysis of m6A regulators (**C**) and EMT regulators (**D**) in normal and ovary cancer samples. Relationship among m6A regulators (**E**) or EMT regulators (**F**) were displayed by the package “ggpubr” and “ggExtra” based on “ggplot2” in R software (Version 3.6.3). A fork indicated that the correlation between two regulators did not accord with P < 0.05.

To identify the critical roles of EMT and m6A regulators in the initiation and development of ovarian cancer, we conducted a comprehensive investigation of the transcription patterns of 19 EMT regulators and 22 m6A regulators. The transcriptional characterization of EMT (Fig. 1A, C) and m6A regulators (Fig. 1B, D) were represented using heatmaps and violin plots respectively. The results showed significant differences in transcription patterns between ovarian cancer and normal samples. To further investigate the interactions between EMT and m6A regulators in ovarian cancer samples, we conducted additional analysis to examine the correlations among these factors (Fig. 1E, F). Our findings revealed a statistically significant correlation.

### Correlation between EMT and m6A regulators

**Figure 2:**
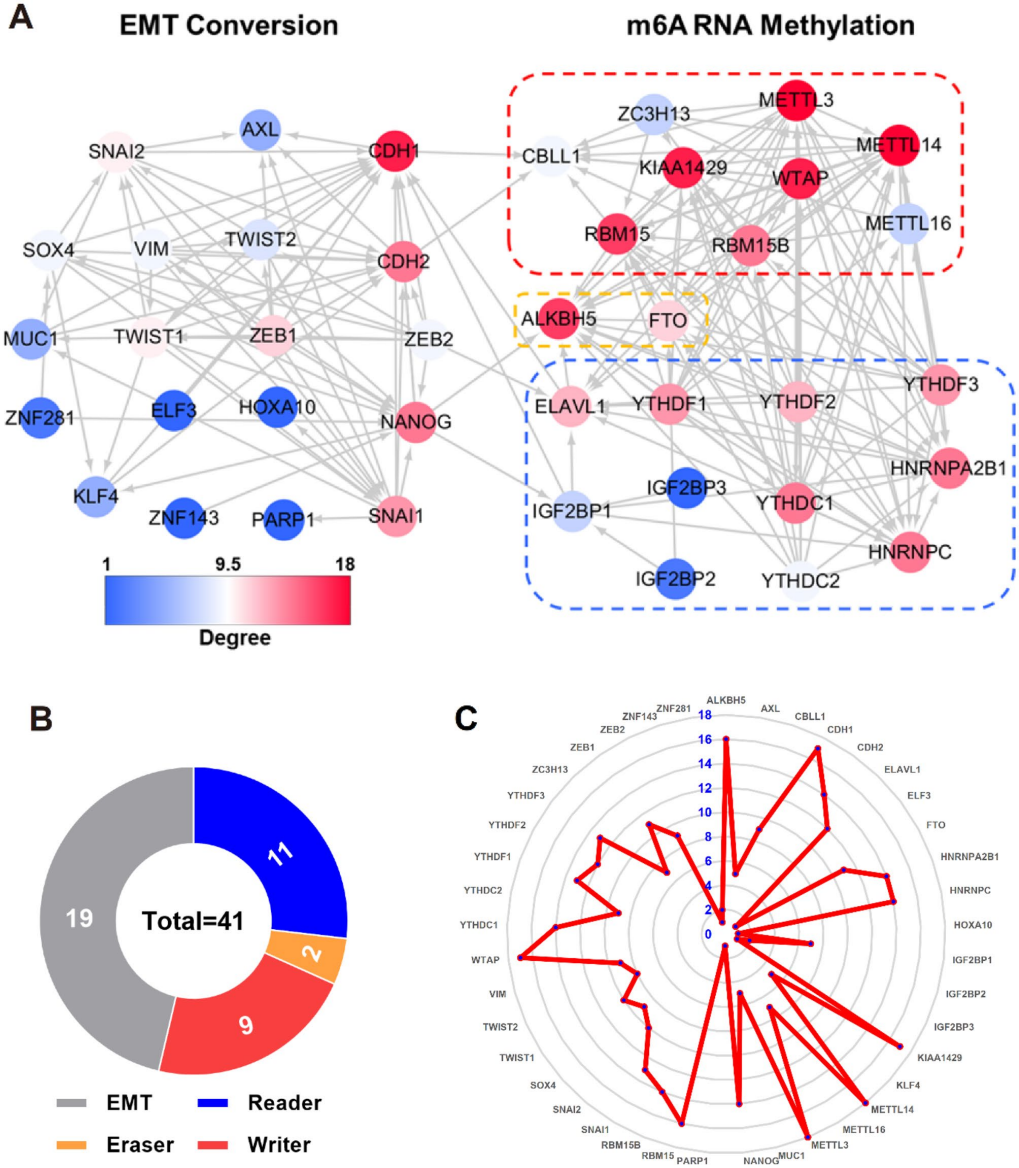
Correlations landscape among EMT and m6A regulators. (**A**) The protein–protein interactions among EMT and m6A regulators were acquired via STRING (version 11.5, https://www.string-db.org/) and visualized using Cytoscape (Version 3.6.1). (**B**) The composition summary of EMT and m6A regulators. (**C**) Radar map of the number of EMT and m6A regulators.

A total of 41 individuals were involved in this study, including 19 EMT regulators and 22 m6A regulators (9 writers, 11 readers, and 2 erasers), as shown in Fig. 2B. The PPIs network (Fig. 2A) and Radar plot (Fig. 2C) depicted a closely related mutual regulation between EMT and m6A regulators. In addition, Table S1 summarizes the topology parameters of the PPI network, including Betweenness centrality (Figure S2A), Avg. clustering coefficient (Figure S2B), Topology coefficient (Figure S2C), Closeness centrality (Figure S2D), Frequency of path length (Figure S2E), Avg. neighborhood connectivity (Figure S2F), Stress centrality (Figure S2G), Frequency of neighbors (Figure S2H), and Distribution of the node degree (Figure S2I).

### Investigating the EMT and m6A prognostic signatures

**Figure 3:**
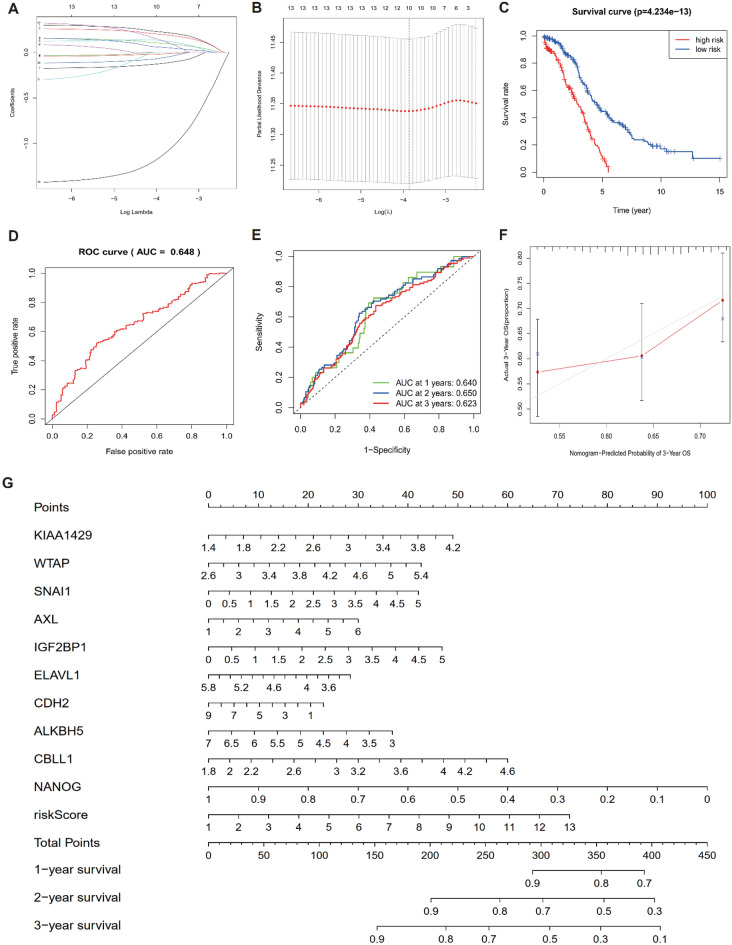
Construction of EMT and m6A prognostic signature. (**A**) The LASSO analysis confirmed the EMT and m6A regulators relevant to prognosis. (**B**) The optimal coefficients determined via multiple verification. (**C**) overall survival rate between low risk (blue) and high risk (red) group. (**D**) ROC curve for predicting overall survival. (**E**) Time-dependent ROC curve for predicting overall survival in ovary cancer samples at one (green), two (blue), and three (red) year. (**F**) Calibration maps used to predict the overall survival at 3 years in patients with ovary cancer. (**G**) The Nomogram to furcate the survival time of ovary cancer.

To analyze the prognostic effects of individual EMT and m6A regulators, we performed univariate independent prognostic analysis on the transcriptional characterization of these regulators (Table S2). The study findings indicated that out of the 41 regulators, 7 (*P* < 0.1) were significantly associated with overall survival, as illustrated in Figure S3A-B. Of these seven regulators, namely KIAA1429, WTAP, SNAI1, AXL, IGF2BP1, ZEB1, and ELAVL1 (except ELAVL1), all had been previously identified as risk regulators with a Hazard Ratio > 1. Subsequently, mutual regulation factors including CBLL1, CDH1, CDH2, NANOG, IGF2BP1, ALKBH5, and ELAVL1 were incorporated to conduct LASSO regression analysis (Figure S3C). Ten prognostic signatures, including KIAA1429, WTAP, SNAI1, AXL, IGF2BP1, ELAVL1, CBLL1, CDH2, NANOG and ALKBH5, were confirmed through LASSO regression analysis (Fig. 3A, B). We combined the expression level with the coefficients (Table S3) of each signature regulator to calculate the risk score. After the analysis, the ovarian cancer samples were categorized into high-risk and low-risk groups based on the median risk score. The Kaplan–Meier curve revealed a significantly higher survival rate for the low-risk group compared to the high-risk group (*P* = 4.234e−13), indicating that the risk score was a reliable prognostic indicator (Fig. 3C). Therefore, we compared the expression and survival characteristics of prognostic signatures (Figure S3). It is evident that there were significant differences in the transcription patterns of prognostic signatures between high-risk and low-risk patients (Figure S4A). Risk curves and scatter plots were used to illustrate the risk scores and survival rates for all ovarian cancer patients, revealing that the mortality or lifetime of low-risk patients was notably lower than that of high-risk patients (Figure S4B-C). The ROC analysis was carried out using the risk score to determine the sensitivity and specificity of survival prediction. The accuracy of the risk model was evaluated by calculating the area under the curve (AUC) of the ROC curve. The AUC value of 0.648, as shown in Fig. 3D, indicated that the constructed risk model was accurate. Moreover, the accuracy of prognostic signatures was assessed by calculating the overall survival of ovarian cancer patients at 1, 2 or 3 years using time-dependent ROC curves (Fig. 3E). The AUC values for 1 year (0.640), 2 years (0.650), and 3 years (0.623) showed that prognostic signatures were highly accurate in predicting overall survival. Overall, the aforementioned analysis identified ten regulators as a prognostic signature for ovarian cancer. To enhance the quantitative approach for superior outcome prediction, a nomogram was established for prognostic signatures associated with ovarian cancer. The calibration curve obtained was in close agreement with the ideal model, as shown in Fig. 3F. A higher total point on the nomogram (Fig. 3G) indicates a worse survival outcome.

### Effect of immunocyte infiltration in tumor microenvironment on prognostic characteristics

We further investigated the relationship between immunocyte connection, risk score, and infiltration levels in various risk subtypes to evaluate the impact of ten prognostic regulators on the immune microenvironment of ovarian cancer. The risk score showed positive correlation with B cell (*p* = 0.669) (Figure S5A), NK cells (*P* = 0.313) (Figure S5D), cancer-associated fibroblasts (*p* = 5.974E−07) (Figure S5E), endothelial cells (*p* = 0.025) (Figure S5F) and macrophages (*p* = 8.517E−05) (Figure S5G), while CD4 + T cells (*p* = 0.074) (Figure S5B), CD8 + T cells (*p* = 0.327) (Figure S5C) and uncharacterized cells (*p* = 6.363e−08) (Figure S5H) were all negatively associated with the risk score. Meanwhile, we also investigated the correlation between the risk score and tumor microenvironment-related cell types and infiltration levels to evaluate its impact on the tumor microenvironment. Our findings revealed a negative association between tumor purity (*p* = 2.197E−11) (Figure S5I) and the risk score, while stromal cell score (*p* = 1.096E−13) (Figure S5J), adipocytes (6.315E−06) (Figure S5K), and microenvironment score (6.215E−11) (Figure S5L) were positively correlated with the risk score. These results provide further evidence for the significant relationship between modulator-based prognostic signatures and the immune microenvironment of ovarian cancer.

### Verification and evaluation of immune cluster for ovarian cancer

**Figure 4:**
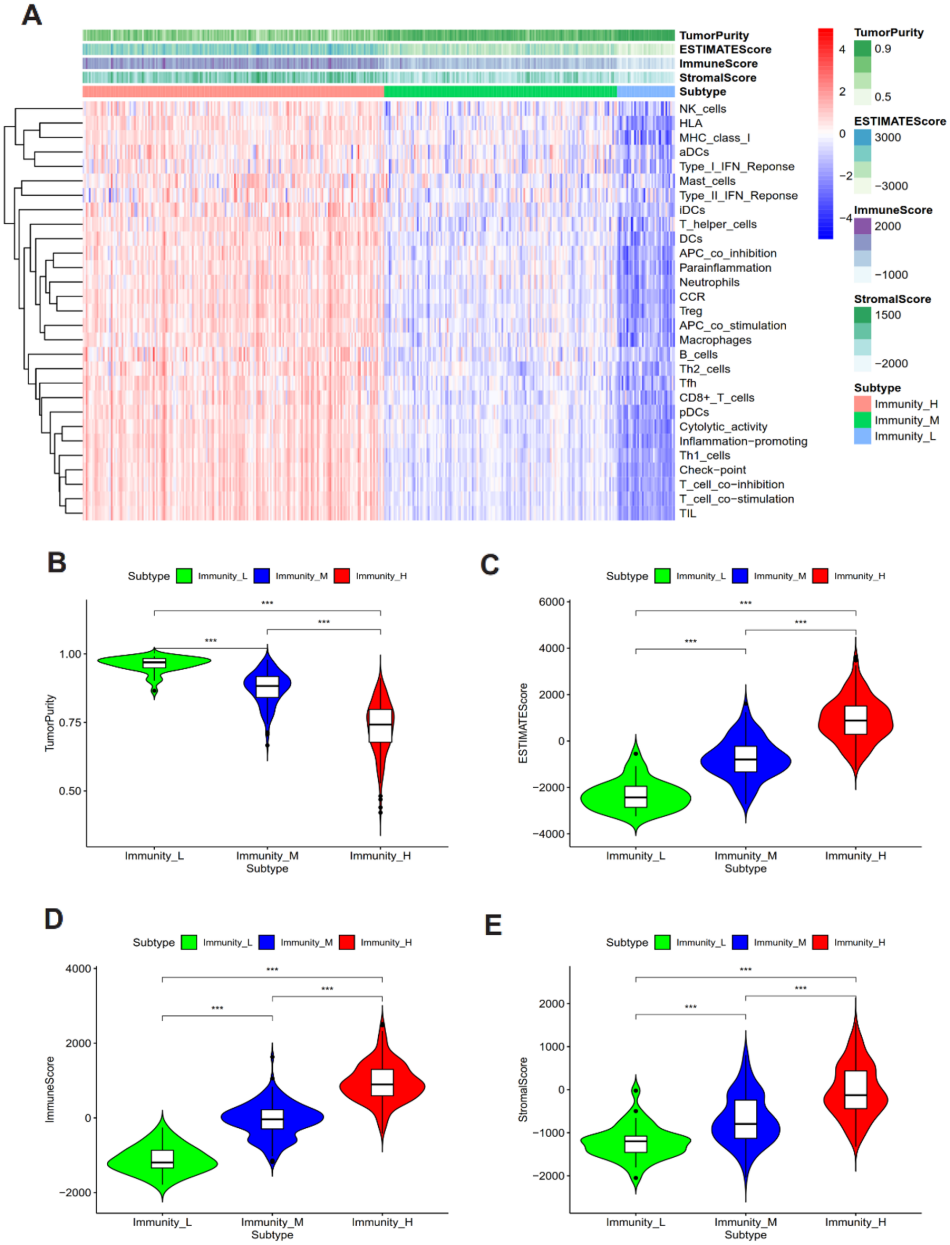
Identification of different immunocyte subtypes infiltration levels in ovarian cancer (**A**) Use the package “pheatmap” in R software (version 3.6.3) to compare the immunoprofiles of ovarian cancer with high, medium, and low levels of immune cell infiltration. The distribution of Tumor Purity (**B**), ESTIMATE Score (**C**), Immune Score (**D**), and Stromal Score (**E**) among three clusters.

**Figure 5:**
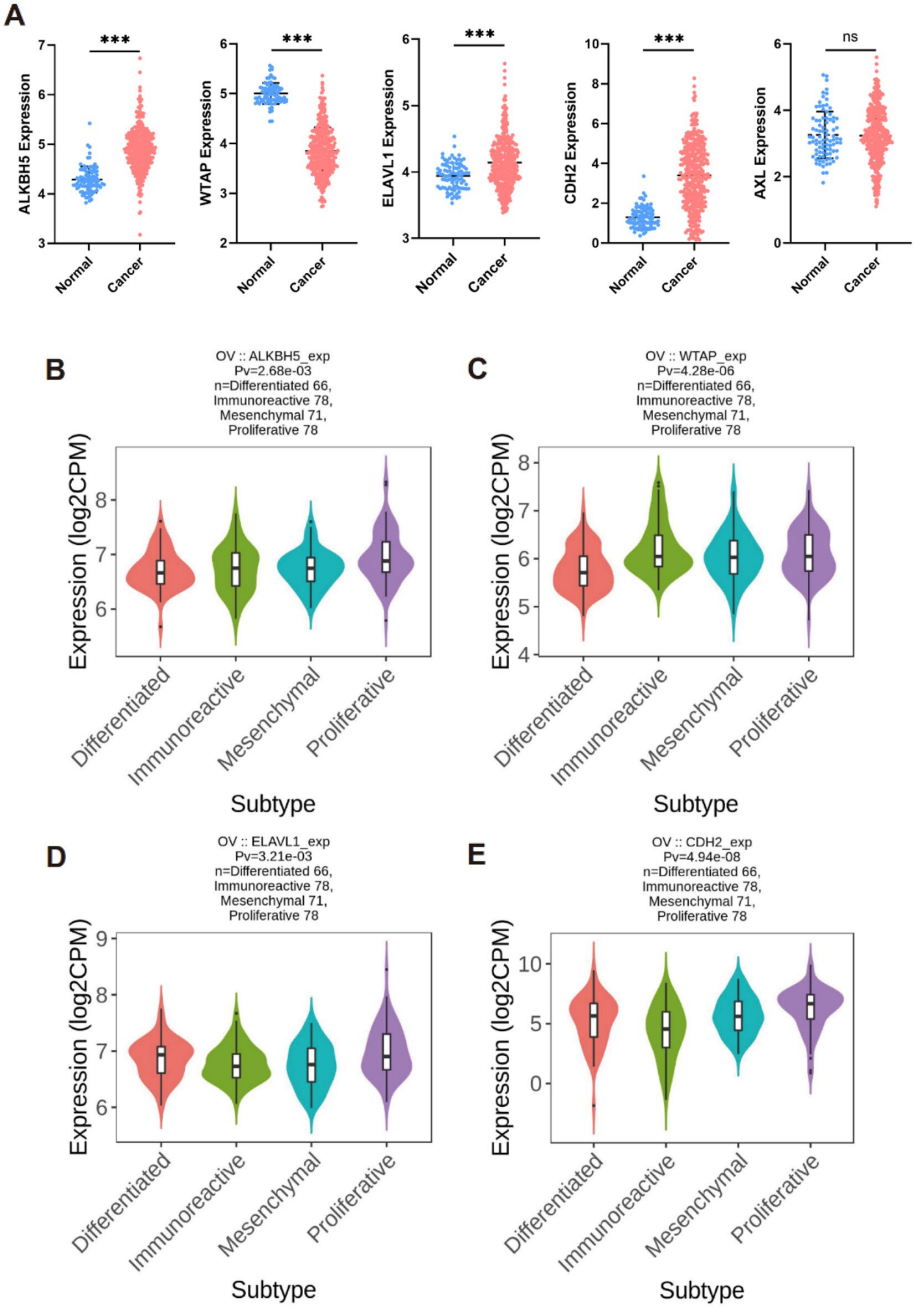
The key signatures expression landscape and molecular subtypes. (**A**) The expression of key signatures. Associations between ALKBH5 (**B**), WTAP (**C**), ELAVL1 (**D**), CDH2 (**E**), expression and molecular subtypes across ovary cancer were calculated derived from TISIDB database.

The ssGSEA algorithm was utilized to assess the degree of immunocyte infiltration in cases of ovarian cancer. Subsequently, the abundance of 29 immune-related cells or types in ovarian cancer samples was determined to evaluate the corresponding score. Through the use of an unsupervised clustering algorithm (with a truncation value of 1.0), ovarian cancer samples were classified into three clusters based on their respective immune infiltration scores, namely high (n = 193), medium (n = 149), and low (n = 37) immunocyte infiltration clusters (Fig. 4A). Secondly, to confirm the accuracy of the clustering mentioned above, we utilized the ESTIMATE algorithm to compute Tumor Purity, ESTIMATE Score, Immune Score, and Stromal Score based on the expression levels of relevant molecules in ovarian cancer. The outcomes revealed that the Tumor Purity of the high immunocyte infiltration cluster was lower than the other two clusters, whereas the Stromal Score, Immune Score, and ESTIMATE Score were inversely related (Fig. 4A). The results regarding tumor purity (Fig. 4B), ESTIMATE Score (Fig. 4C), Immune Score (Fig. 4D), and Stromal Score (Fig. 4E) in the three distinct immune clusters were presented as violin plots, indicating consistent findings. Additionally, the Kaplan–Meier curve demonstrated that patients with low immunocyte infiltration had a lower survival rate, with a significant statistical difference observed among the three clusters (*P* = 0.011) (Figure S6A). To analyze the immune cell types, the CIBERSORT algorithm was employed. The results showed that out of the 22 immune cell types, only memory B cells, plasma cells, and CD8 + T cells exhibited notable variations across three immune clusters. In the high immune clusters, plasma cells and CD8 + T cells were found to be present in high proportions, whereas memory B cells were present in an opposite proportion (Figure S6B). We utilized box plots to visualize the expression differences in prognostic signatures among low, medium, and high immunocyte clusters. Furthermore, our findings indicated that the transcriptional levels of WTAP, SNAI1, IGF2BP1, ELAVL1, CDH2, AXL, and ALKBH5 varied significantly across different immune cell infiltration clusters (Figure S6C). These results enable us to identify seven immunological features associated with ovarian cancer. The expression landscape of key prognosis-related immunological signatures was obtained through Fig. 5A, which showed that ALKBH5, WTAP, ELAVL1, and CDH2 were the primary immune signatures associated with the prognosis of ovarian cancer, with AXL expression levels being similar in both ovarian cancer and normal tissues.

### Correlations among expressions of key signatures with immunomodulators and molecular subtypes of ovarian cancer

To further investigate the influence of key signatures on the immune response of ovarian cancer, we calculated the correlations between signature expression and immunomodulators or methylation, as shown in Figure S7. The findings indicated that ALKBH5, ELAVL1, and CDH2 exhibited a negative correlation with Immunoinhibitors, Immunostimulators, and MHC molecules, as depicted in Figure S7A-C. Conversely, WTAP displayed a positive association with the immunomodulator in ovarian cancer, as shown in Figure S7A-C. Additionally, significant correlations were observed between the methylation of ALKBH5, WTAP, ELAVL1, CDH2 and immunoinhibitors (Figure S7D), immunostimulators (Figure S7E), and MHC molecules (Figure S7F) in ovarian cancer using Spearman’s correlation analysis. Simultaneously, we calculated associations between the expression of key signatures and molecular subtypes in ovarian cancer using the TISIDB database. Our results showed that there were significant associations between the expression of ALKBH5 (Fig. 5B), WTAP (Fig. 5C), ELAVL1 (Fig. 5D), and CDH2 (Fig. 5E) and molecular subtypes (including differentiated, immunoreactive, mesenchymal, and proliferative).

### Expression of Immune checkpoint molecules and clinical benefit prediction for immunotherapy of ovarian cancer

**Figure 6:**
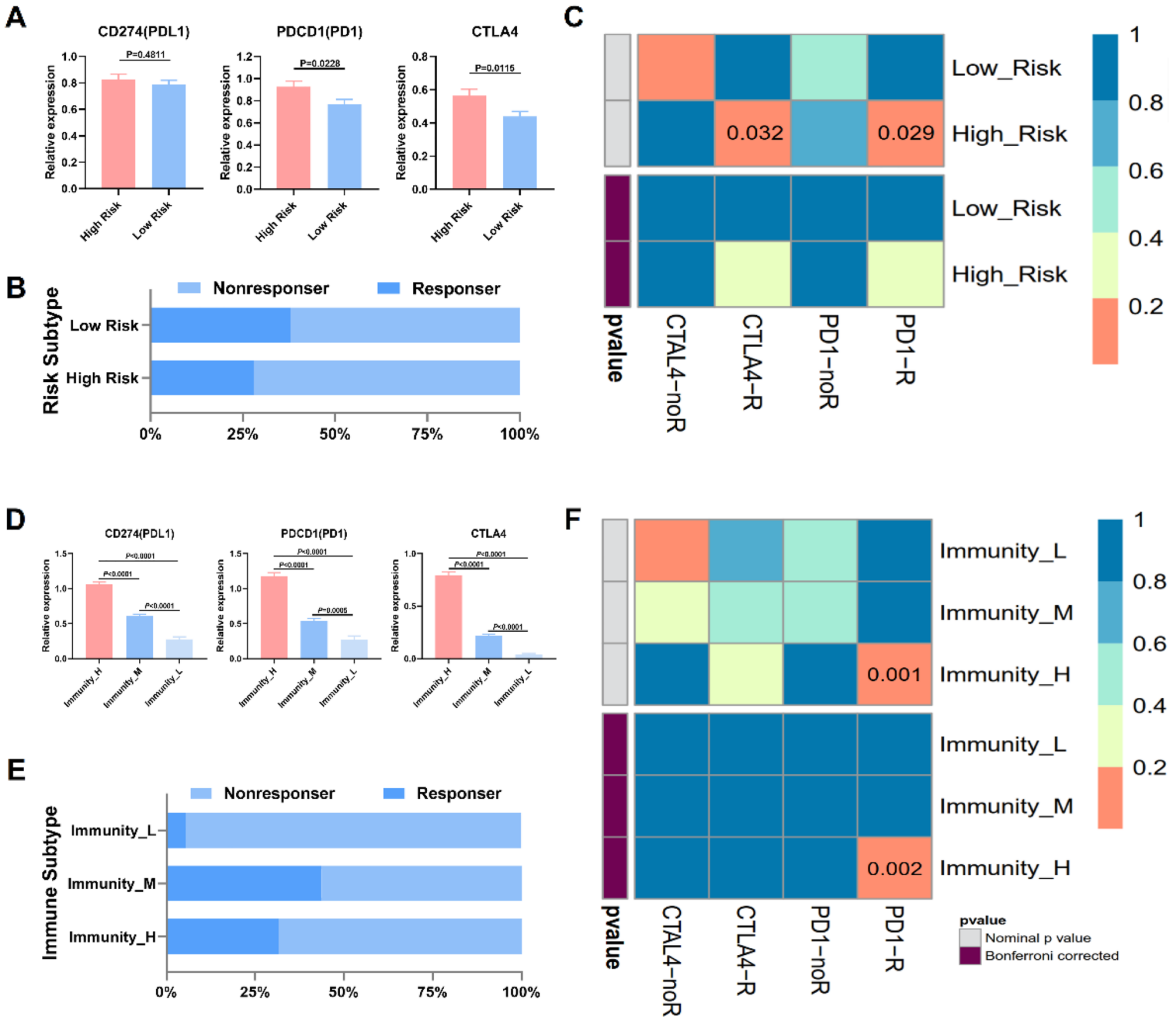
Immune checkpoint molecular expression and clinical benefit prediction in ovarian cancer. (**A**) Expression levels of PDL1, PD1 and CTLA4 between high risk and low risk. (**B**) The proportion of responders to immunotherapy between high and low risk subtype. (**C**) Subclass mapping analysis for predicting the likelihood of a response to immune checkpoint block therapy for the risk subtypes. (**D**) Expression levels of PDL1, PD1 and CTLA4 among different immune subtypes. (**E**) The proportion of responders to immunotherapy among immunity-H, immunity-M and immunity-L subtype. (**F**) Subclass mapping analysis for predicting the likelihood of a response to immune checkpoint block therapy for the immune subtypes. R, response to immune checkpoint block therapy (Bonferroni corrected *p* value < 0.05); noR, no response to immune checkpoint block therapy (Bonferroni corrected *p* value > 0.05).

The levels of expression for PDL1, PD1, and CTLA4 showed a descending trend between high and low-risk groups (Fig. 6A). To predict the likelihood of ovarian cancer patients responding to immunotherapy, Tumor Immune Dysfunction and Exclusion (TIDE) was utilized. The results demonstrated that the proportion of response was highest in the high-risk group (42.85%) compared to the low-risk group (24.87%) (Fig. 6B). Subclass mapping analysis was utilized to predict the effectiveness of immune checkpoint block therapy for ovarian cancer patients categorized as high or low risk (Fig. 6C). Notably, patients at high risk exhibited encouraging responses to anti-PD-1 therapy, whereas those at low risk did not show any response to anti-PD-1 therapy. Based on the aforementioned analyses, it is suggested that patients at high risk may exhibit greater sensitivity to immune checkpoint block therapy. Additionally, the expression levels of PDL1, PD1, and CTLA4 also displayed a decreasing trend across different immune subtypes (Fig. 6D). The response rates to immunotherapy were found to be 31.61% for immunity-H, 43.62% for immunity-M, and 5.41% for immunity-L (Fig. 6E). Subclass mapping analysis revealed that the immunity-H subtype may be particularly responsive to anti-PD-1 treatment (Fig. 6F). All *p*-values were adjusted accordingly.

### Characteristic alteration and synergistic effect of key signatures

**Figure 7:**
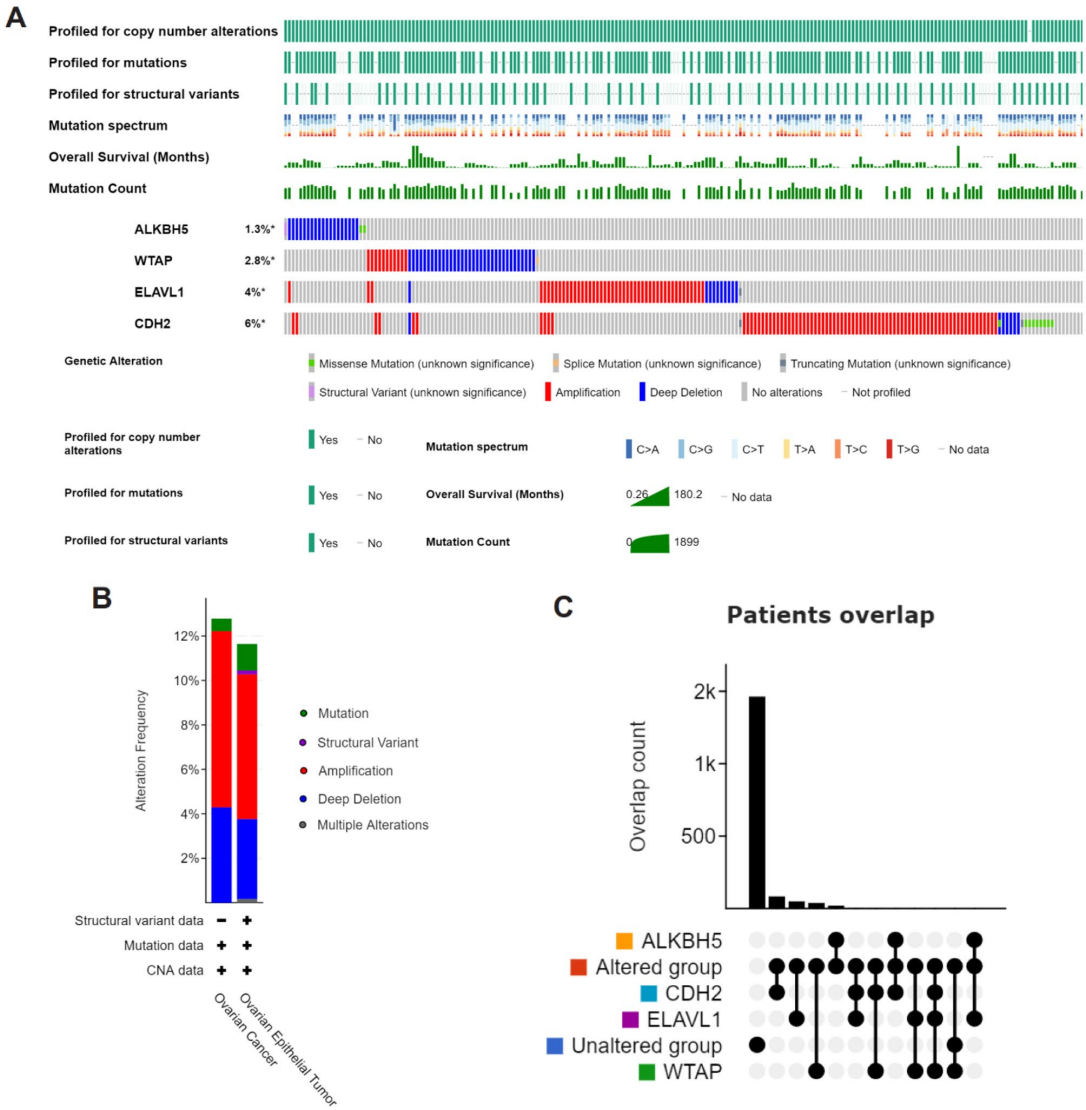
Characteristic alteration of Key signatures. (**A**) The genetic variation, copy number alterations, mutation spectrum, mutations, structural variants, overall survival and mutation count connected with the key signatures were displayed as a visual summary for ovarian cancer samples from the TCGA database via CBioPortal (version 5.3.12, http://www.cbioportal.org/). (**B**) An overview of the alteration of key signatures for ovarian cancer. (**C**) Patients overlap between Altered group and Unaltered group were excluded from patient-level analysis in other tabs.

The cBioPortal analysis confirmed that 205 (12%) of the examined patients had significant changes in key signatures, including copy number alterations, mutation spectrum, mutations, mutation counts, structural variants, and overall survival. Among the alterations, CDH2 showed the most prominent changes (6%) compared to other regulators, including amplification, deep deletion, truncating mutation, and missense mutations (Fig. 7A). Amplification was the most common type of mutation (Fig. 7B). The genes, ALKBH5, WTAP, ELAVL1, and CDH2, which were completely overlapping between the Altered and Unaltered groups, have been excluded from the patient-level analysis in other tabs (Fig. 7C). Simultaneously, to understand the biology behind the key signatures, the protein expression of ALKBH5, WTAP, ELAVL1, and CDH2 in ovarian cancer was analyzed using CPTAC datasets (Figure S8A-D). The results showed that the gene and protein expression patterns of ALKBH5, ELAVL1, and CDH2 were increased in ovarian cancer (Fig. 1A, E and Figure S8A, C, D), while the protein expression of WTAP did not match the gene expression (Fig. 1E, F and S8B), suggesting gene transcription and translation may be involved in its preservation. The study also explored the expression patterns of these genes in various ovarian cancer cell lines using the CCLE database (Figure S8E). Correlation analyses showed a positive correlation between CDH2 and ALKBH5 (Figure S8F, S8I), a significant positive correlation between CDH2 and ELAVL1 (Figure S8H, S8K), and a significant negative correlation between CDH2 and WTAP (Figure S8G, S8K).

## Discussion

Prior evidence has indicated that certain EMT and m6A regulators may play a significant role in the progression of various types of tumors^25,45,46^. However, it is noteworthy that the identical EMT or m6A regulators may exhibit diverse functions in distinct tumors^47–49^. As a crucial mechanism in the progression of ovarian cancer, EMT has been implicated in the development, invasion, metastasis, drug resistance, and recurrence of tumors^12^. The onset of EMT is orchestrated by multiple transcriptional regulators, including CDH1, CDH2, ZEB1, ZEB2, SNAI1, SNAI2, TWIST1, TWIST2, VIM, KLF4, AXL, NANOG, ZNF143, ZNF281, MUC1, PARP1, SOX4, ELF3, and HOXA10^50^. An increasing number of studies have validated that cells undergoing an intermediate transition state during EMT transformation acquire more robust abilities in invasion-migration and anti-apoptosis^51^. Simultaneously, m6A methylation, a dynamic and reversible RNA modification process, is controlled by a complex interplay of m6A methyltransferase complex (writers), m6A demethylase (erasers), and m6A reading proteins (readers) from catalytic formation to functional realization^20^. Being the most prevalent mRNA modification, m6A modification impacts tumor occurrence and progression, particularly in terms of self-renewal, differentiation, apoptosis, invasion and metastasis, drug resistance, immunosuppression, and other events that involve tumor stem cells^29^. Hence, the molecules that play a crucial role in m6A modification are anticipated to serve as potential molecular targets for cancer diagnosis, treatment, prognostic outcome, and drug development.

As most studies have focused solely on EMT or m6A regulators^52^, the co-interaction among multiple EMT and m6A regulators in tumors remains not fully understood. In summary, comprehending the influence of EMT and m6A-mediated immunological signatures associated with different prognoses on ovarian cancer development will aid in further clarifying the impact of the immune microenvironment on prognosis. This, in turn, can help in developing more effective immunotherapy strategies.

In our study, we initially identified ten prognostic signatures, namely KIAA1429, WTAP, SNAI1, AXL, IGF2BP1, ELAVL1, CBLL1, CDH2, NANOG, and ALKBH5, through LASSO regression analysis, which comprehensively assessed their impact on immune infiltrating signatures and final prognostic outcome. Subsequently, we used the ssGSEA algorithm and overall survival analyses to identify the key prognosis-related immunological signatures in ovarian cancer, which included WTAP, ELAVL1, CDH2, and ALKBH5. Once again, the relevant signatures, combined with related immune response and genetic alteration, have demonstrated that the features of EMT regulator CDH2 are significantly associated with m6A regulators ALKBH5, ELAVL1, and WTAP, indicating a synergistic effect on the occurrence and development of ovarian cancer.

CDH2, ALKBH5, ELAVL1, and WTAP are the key regulators that exert a critical impact on the development and prognosis of ovarian cancer. While CDH2, a member of the cadherin superfamily, is typically expressed in neuroectoderm and organs from mesoderm, it is not expressed in normal epithelial tissues^53^. However, when CDH2 is expressed in epithelial cells, it alters the morphology and biological function of the cells, transforming them into mesenchymal cells with increased migration ability. This process is known as EMT^54^. The EMT process leads to cytoskeleton remodeling, reduced intercellular connectivity and adhesion, altered cell polarity, and increased invasion and migration. Consequently, the abnormal expression of CDH2 can enhance the migration and invasion ability of tumor cells, promote cell–cell interaction, and play an essential role in tumor progression and metastasis^55^. Moreover, several studies have confirmed the close association of m6A regulatory factors ALKBH5, WTAP, and ELAVL1 with the pathological process of tumor invasion and metastasis. For instance, in vitro studies have demonstrated that the down-regulation of ALKBH5 can inhibit the growth and invasion of endometrial cancer cells^56^. Studies have revealed that WTAP can act as an oncogene and facilitate the progression of malignant tumors in several cancers, including colorectal cancer and renal cell carcinoma^57,58^. Additionally, WTAP is located on human chromosome 6Q25.3, which has been linked to ovarian cancer^59^. As for ELAVL1, it is involved in multiple biopathological processes and is therefore closely associated with the occurrence and development of various cancers^60^.

In summary, the coordinated regulation of m6A modification and EMT modulators may play a crucial role in the progression or evolution of tumors. Furthermore, other experimental evidence has shown that m6A regulator METTL3 can enhance the expression of EMT regulator AXL, thereby triggering EMT. Additionally, high expression of METTL3 has been linked to poor survival prognosis in ovarian cancer patients^61,62^. Another study has shown that the inhibition of m6A regulator METTL14 in malignant progression may be partially reliant on the SOX4-mediated EMT process^63^. Hence, it is evident that m6A modification and the biological process of EMT may have a synergistic regulatory effect on the occurrence and development of tumors.

Furthermore, it is essential to take note of the limitations of this study, including potential bias in sample selection and incomplete clinical characteristics of the samples. As a result, additional relevant experimental studies will be necessary to uncover the correlation between m6A modification and EMT regulators involved in the physiological and pathological mechanisms of tumors, both in vitro and in vivo.

**Figure 8:**
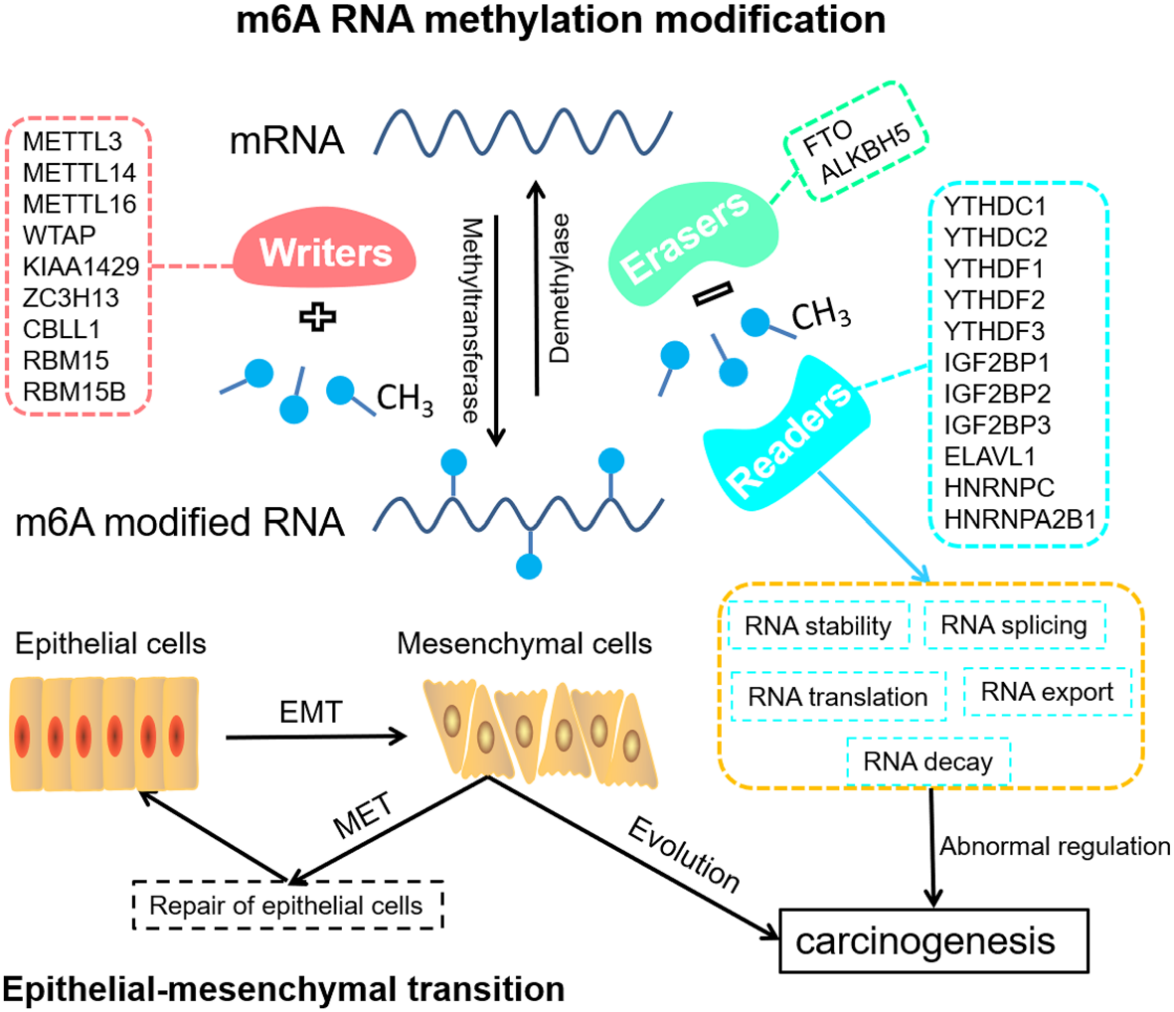
Outline for the synergistic effect on the carcinogenesis between EMT process and m6A modification.

To summarize, the key regulators have been identified as crucial molecules with prognosis-related immunological signatures for the development of ovarian cancer. The transcription pattern of CDH2 was found to be positively correlated with ALKBJ5 and ELAVL1, while negatively correlated with WTAP, indicating that m6A modification and EMT process have a synergistic effect on malignant transformation, cancer occurrence, and development outcome (Fig. 8), particularly for ovarian cancer. In essence, our studies provide a new perspective for predicting the prognosis and survival of ovarian cancer patients based on the synergistic regulation of m6A and EMT regulatory factors in ovarian cancer transcriptional patterns. This could lead to a novel research strategy for the diagnosis, immunotherapy, and prognosis detection of ovarian cancer.

### Supplementary Information

Supplementary Information.

## Data Availability

The datasets for this study are available in the TCGA (https://portal.gdc.cancer.gov) and GETx datasets (https://www.gtexportal.org/home/datasets). Meanwhile, all methods were carried out in accordance with relevant guidelines and regulations.

## Abbreviations

EMT: Epithelial-mesenchymal transformation
m6A modification: N6-methyladenosine modification
TCGA: The Cancer Genome Atlas
GTEx: Genotype tissue expression
ssGSEA: Single sample gene set enrichment analysis
LASSO regression: Least absolute shrinkage and selection operator regression
PPIs: Protein-protein interactions
CPTAC: Clinical Proteomic Tumor Analysis Consortium
GEPIA2: Gene expression profiling interactive analysis 2
CCLE: Cancer Cell Line Encyclopedia
