# Supplementary material for: The synergistic effect of EMT regulators and m6A modification on prognosis-related immunological signatures for ovarian cancer
Source: Sci Rep. 2023 Sep 8;13:14872. doi: 10.1038/s41598-023-41554-y (PMC10491820; doi:10.1038/s41598-023-41554-y)
Supplement: Supplementary file 1 — Supplementary Information. [file 41598_2023_41554_MOESM1_ESM.docx]

**Supplementary**

**
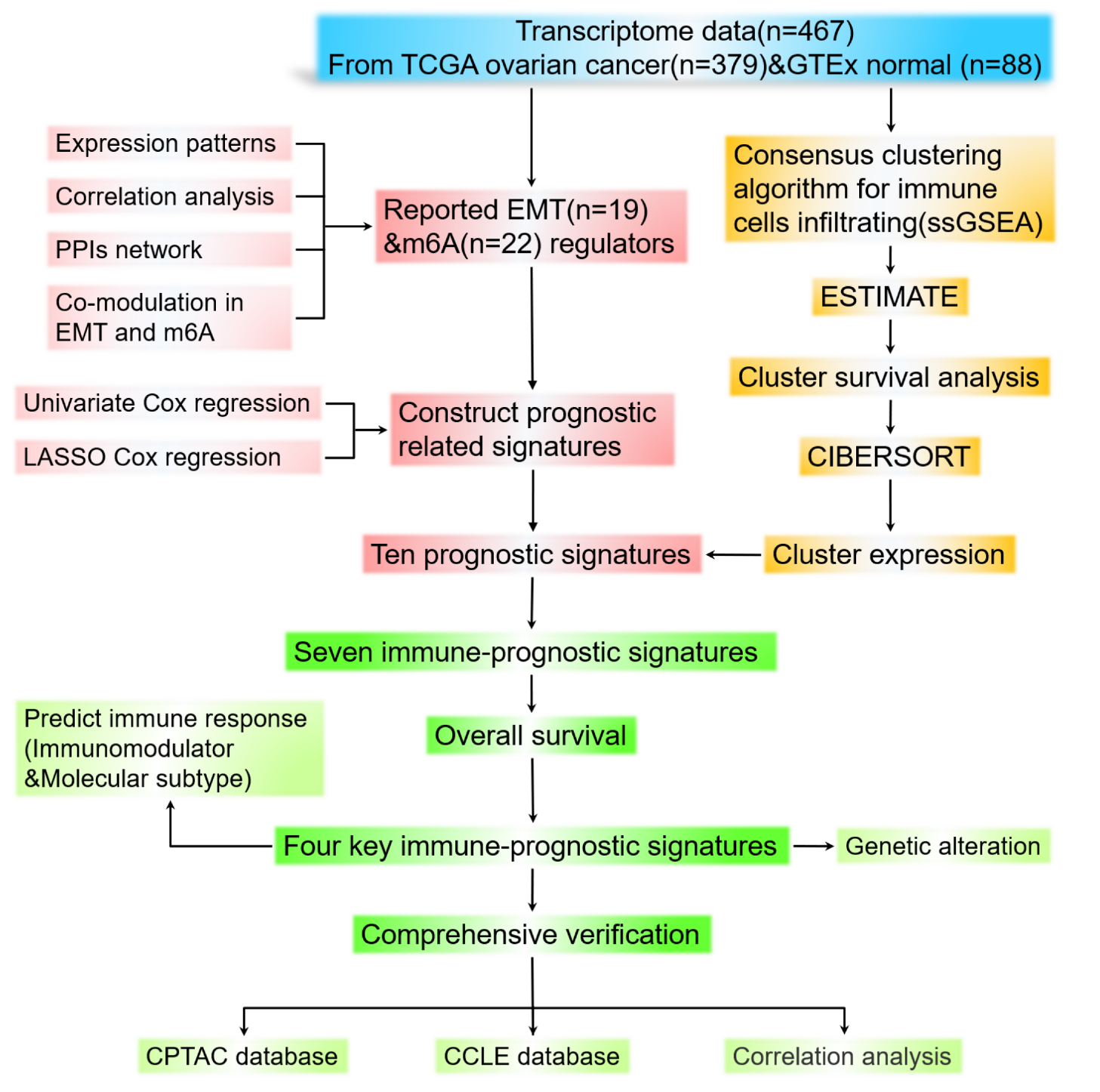
**

**Figure S1: Workflow chart of study design and analysis.**

**
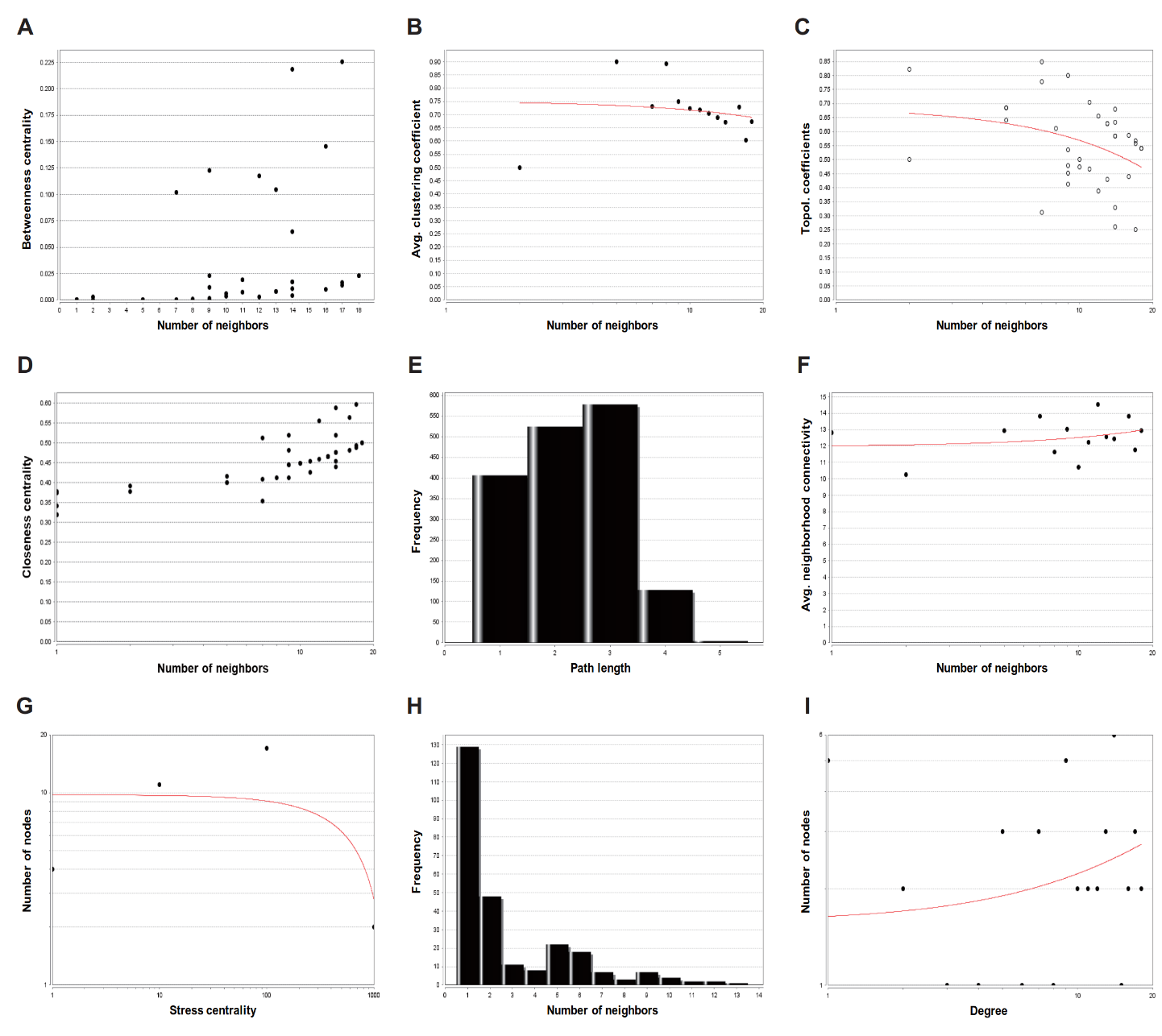
**

**Figure S2: Topology parameters for EMT and m6A modification backbone network.** (A) Betweenness centrality. (B) Avg. clustering coefficient. (C) Topology coefficient. (D) Closeness centrality. (E) Frequency of path length. (F) Avg. neighborhood connectivity. (G) Stress centrality. (H) Frequency of neighbors. (I) Distribution of the node degree.

**
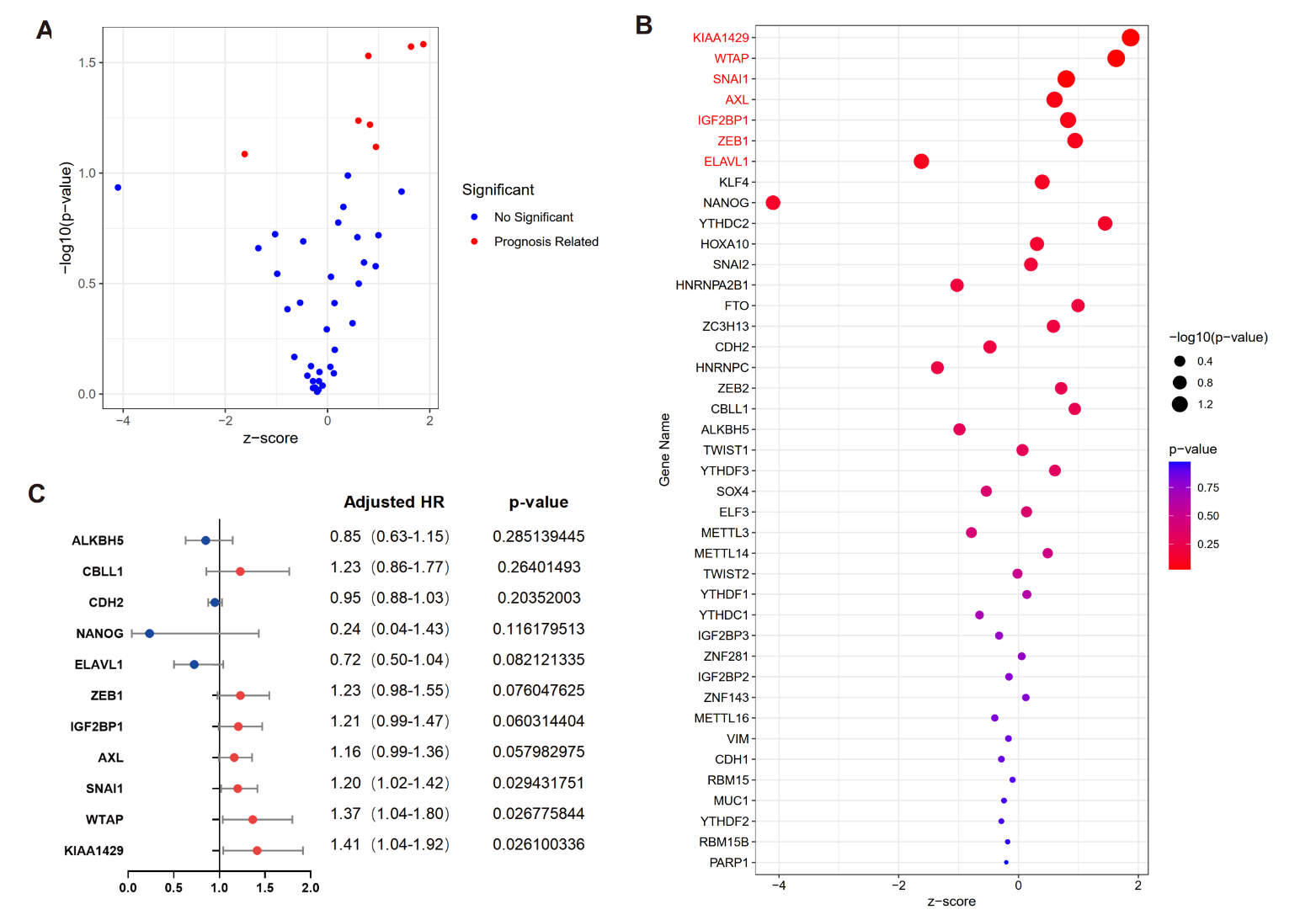
**

**Figure S3. The selection of prognosis characteristics from EMT and m6A regulators.** (A)Volcano plot and (B) Bubble chart of univariate independent prognostic analysis among EMT and m6A regulators. (C) Forest map of the EMT and m6A regulators was related to prognosis and co-regulation in ovary cancer.


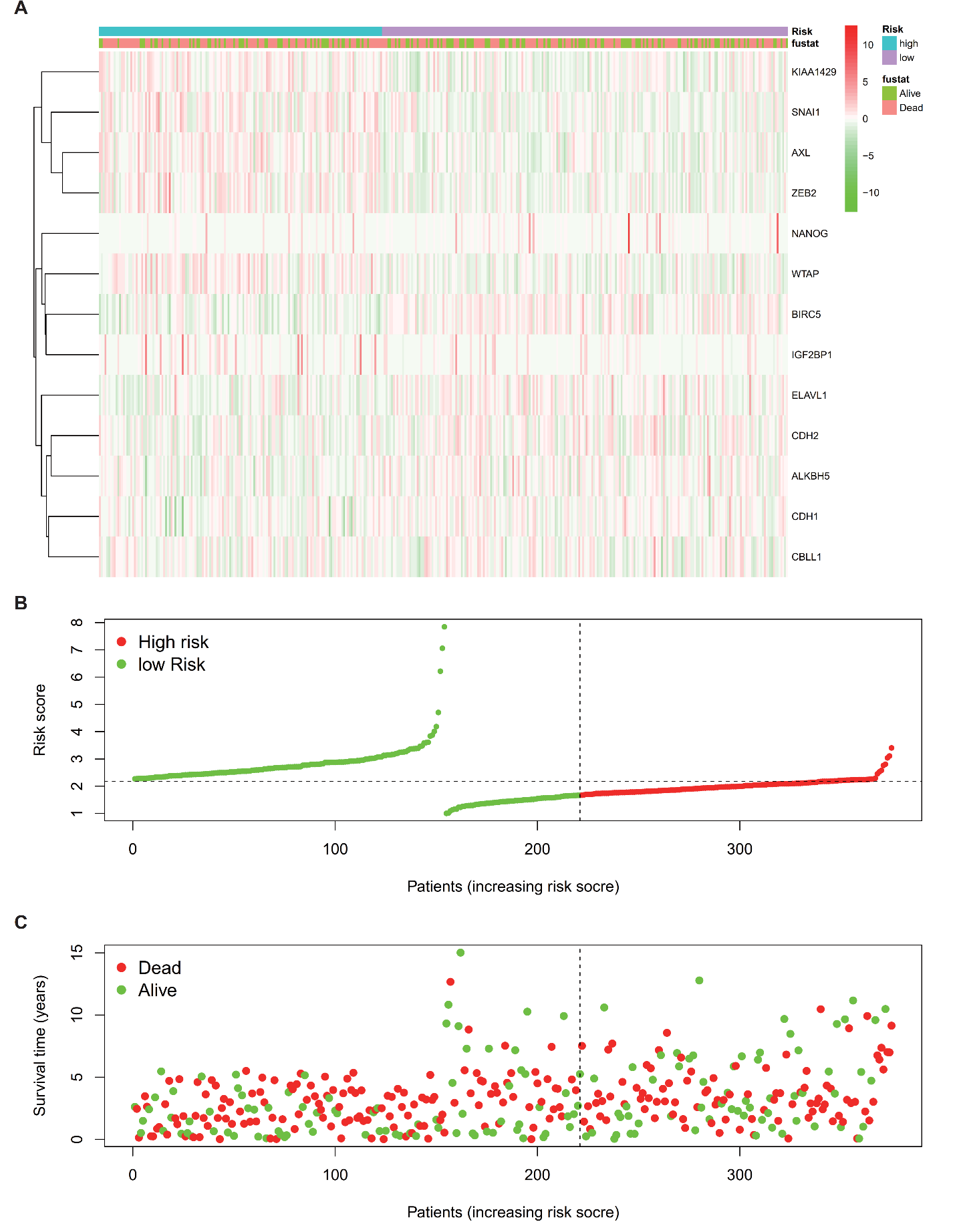


**Figure S4**: The distribution on the expression heatmap (A), risk score (B) and overall survival status (C) were visualized via the package "pheatmap" in R software (Version 3.6.3) for the EMT and m6A signatures in the ovarian cancer dataset.


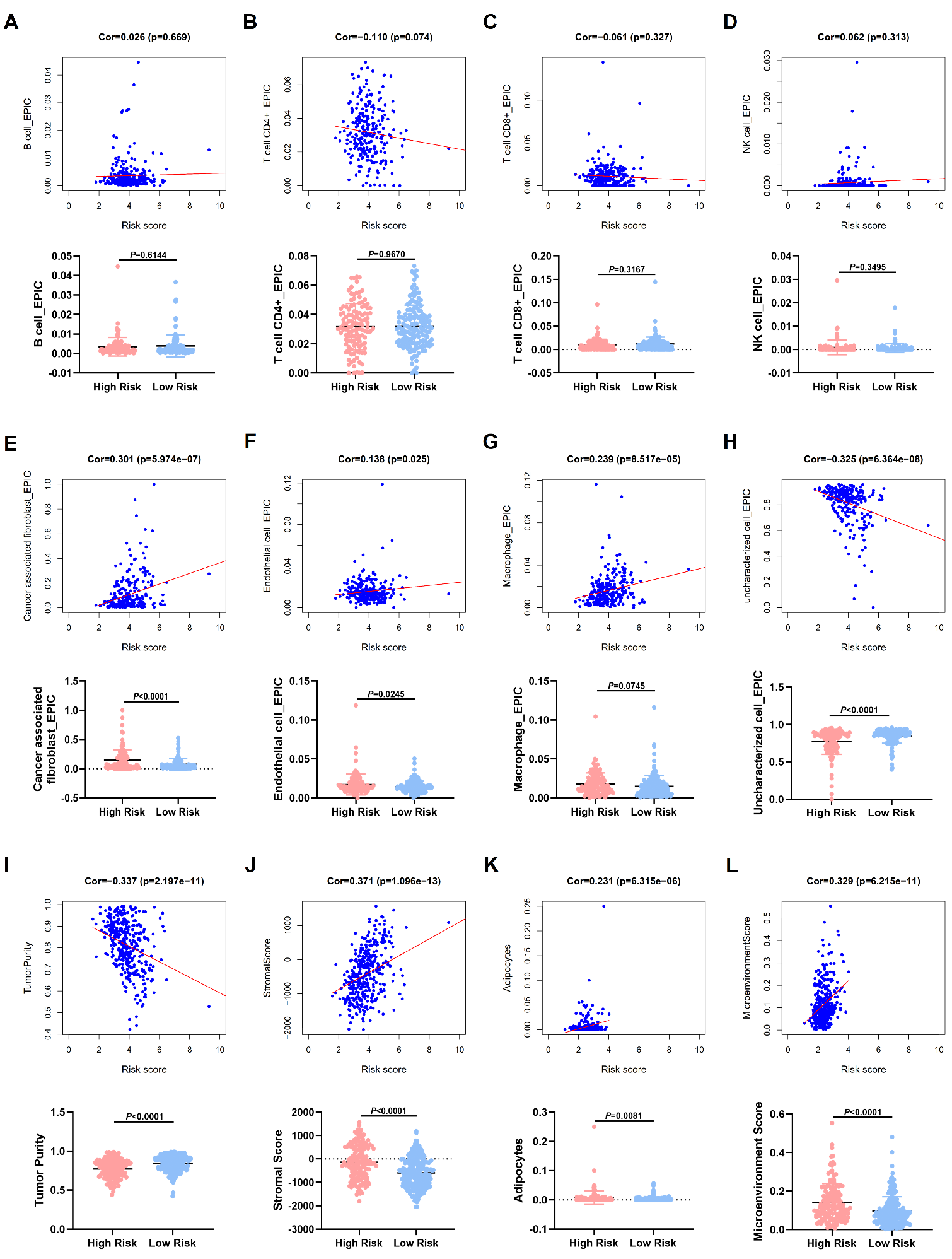


**Figure S5: Effect of risk score on immunocyte infiltration and tumor microenvironment.** Correlation of risk score with immunocyte or tumor microenvironment related cell types and these infiltration levels, including B cells(A), CD4+ T cells(B), CD8+T cells(C), NK cells(D), Cancer associated fibroblast cells(E), endothelial cells(F), macrophages(G), uncharacterized cells(H), tumor purity(I), stromal cell score (J), adipocytes (K) and microenvironment score (L) to evaluate the impact on ovarian cancer prognosis.

**
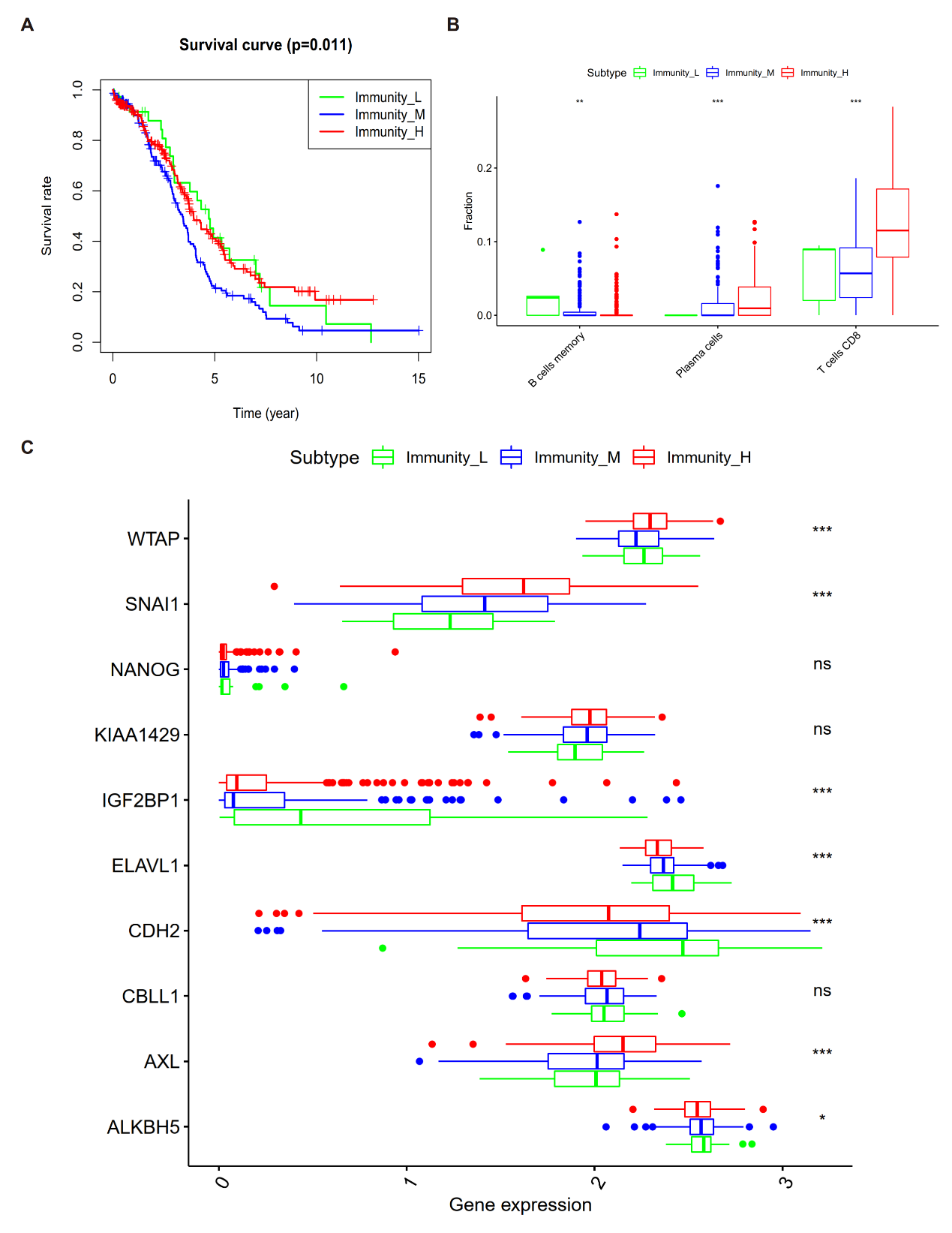
**

**Figure S6: Correlation between immune clustering and overall survival.** (A)Survival rate for low (green), medium (blue) and high (red) immunocyte infiltration cluster. (B) The fraction of immune cell types via CIBERSORT algorithm in low (green), medium (blue) and high (red) immunocyte infiltration cluster. (C)The expression of risk signature regulators was a significant difference among three immunocyte infiltration clusters.

**
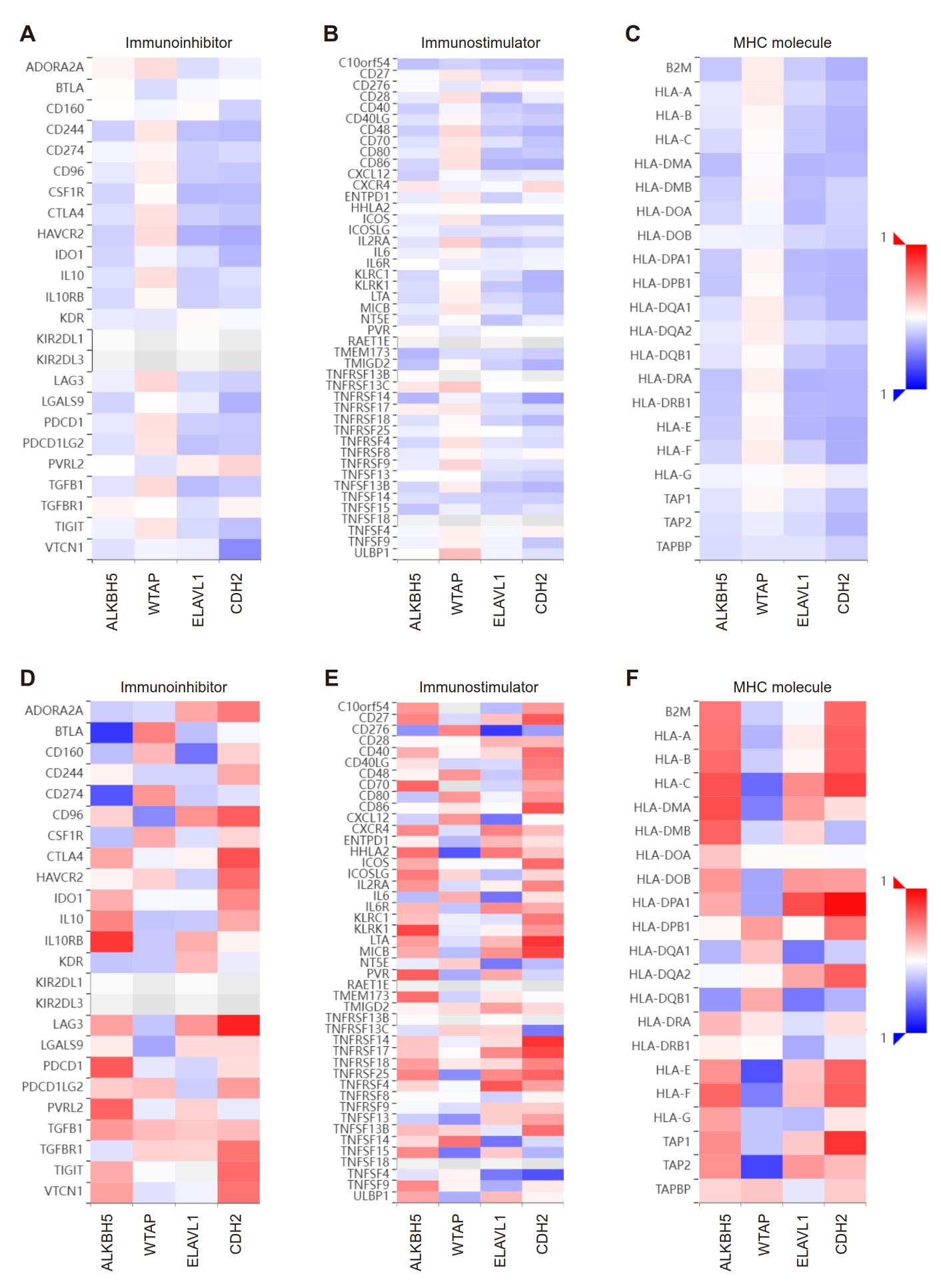
**

**Figure S7: Correlations between key signatures and the Immunomodulator or methylation across ovary cancer.** Spearman relationship for the Immunomodulator and the expression of ALKBH5, WTAP, ELAVL1, CDH2 (A-C) and correlations between methylation of ALKBH5, WTAP, ELAVL1, CDH2 and Immunoinhibitors (D), Immunostimulator (E), and MHC molecules (F) across ovary cancer were calculated derived from TISIDB database (http://cis.hku.hk/TISIDB/).

**
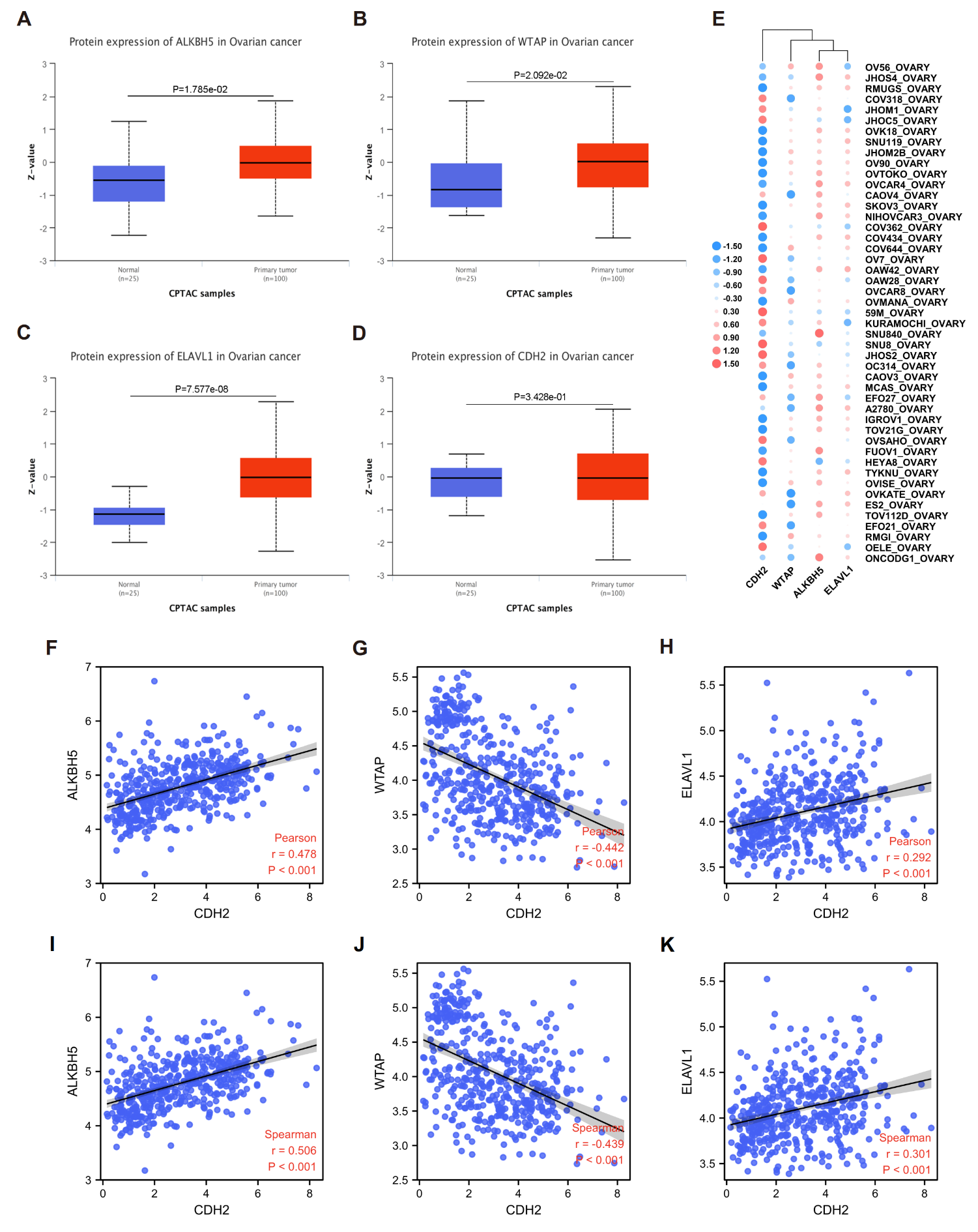
**

**Figure S8:** **Key signatures validation and relationships in EMT and m6A regulators.** The difference protein expression patterns between ovarian cancer and normal samples for ALKBH5(A), WTAP(B), ELAVL1(C), CDH2(D) based on CPTAC dataset. Key signatures expression levels were identified in various ovarian cancer cell lines from CCLE database (E). Pearson correlation analyses of CDH2 and ALKBH5(F), WTAP(G), ELAVL1(H). Spearman correlation analyses of CDH2 and ALKBH5(I), WTAP(J), ELAVL1(K).

| **Supplementary Tables**   \| **TableS1: Topological parameters for EMT and m6A regulators PPIs network** \| \| \| --- \| --- \| \| **Topological parameters** \| **Comprehended values** \| \| Number of nodes \| 41 \| \| Network density \| 0.248 \| \| Network heterogeneity \| 0.529 \| \| Clustering coefficient \| 0.623 \| \| Network diameter \| 5 \| \| Network radius \| 3 \| \| Network centralization \| 0.213 \| \| Shortest paths \| 1640(100%) \| \| Characteristic path length \| 2.268 \| \| Avg. number of neighbors \| 9.902 \|   **TableS2: Univariate Cox regression analyses were used to examine the associations between expression of m6A or EMT regulators and prognosis** | | | | |
| --- | --- | --- | --- | --- | --- | --- | --- | --- | --- | --- | --- | --- | --- | --- | --- | --- | --- | --- | --- | --- | --- | --- | --- | --- | --- | --- | --- | --- |
| **id** | **HR** | **HR.95L** | **HR.95H** | **pvalue** |
| KIAA1429 | 1.413175883 | 1.042015204 | 1.916542165 | 0.026100336 |
| WTAP | 1.365755801 | 1.036512345 | 1.799581952 | 0.026775844 |
| SNAI1 | 1.201089451 | 1.018485394 | 1.416432555 | 0.029431751 |
| AXL | 1.162447738 | 0.994920463 | 1.358183687 | 0.057982975 |
| IGF2BP1 | 1.207101357 | 0.991869477 | 1.469037732 | 0.060314404 |
| ZEB1 | 1.230277073 | 0.97852174 | 1.546804341 | 0.076047625 |
| ELAVL1 | 0.723981263 | 0.503021251 | 1.042001442 | 0.082121335 |
| KLF4 | 1.121815411 | 0.977214218 | 1.287813658 | 0.102553927 |
| NANOG | 0.23518163 | 0.038652649 | 1.430960127 | 0.116179513 |
| YTHDC2 | 1.329147671 | 0.927469491 | 1.904788836 | 0.121179909 |
| HOXA10 | 1.104696955 | 0.967113403 | 1.261853427 | 0.142316409 |
| SNAI2 | 1.084735226 | 0.966383616 | 1.217581187 | 0.167630282 |
| HNRNPA2B1 | 0.841396837 | 0.650341232 | 1.088580276 | 0.188809841 |
| FTO | 1.239935974 | 0.898201645 | 1.711688268 | 0.191101237 |
| ZC3H13 | 1.158734952 | 0.927332828 | 1.447880037 | 0.194902406 |
| CDH2 | 0.949769073 | 0.877242159 | 1.028292225 | 0.20352003 |
| HNRNPC | 0.776567725 | 0.519165986 | 1.161588873 | 0.218374732 |
| ZEB2 | 1.184270007 | 0.885508132 | 1.583831246 | 0.254198985 |
| CBLL1 | 1.229150696 | 0.855797681 | 1.765383883 | 0.26401493 |
| ALKBH5 | 0.849328012 | 0.629534588 | 1.145859314 | 0.285139445 |
| TWIST1 | 1.056808163 | 0.953067445 | 1.171840984 | 0.294585193 |
| YTHDF3 | 1.163859569 | 0.865108104 | 1.565780149 | 0.316062862 |
| SOX4 | 0.93786964 | 0.811210432 | 1.084304919 | 0.386194405 |
| ELF3 | 1.070322349 | 0.917539018 | 1.248546283 | 0.387140171 |
| METTL3 | 0.88878884 | 0.670017359 | 1.178992739 | 0.413477909 |
| METTL14 | 1.140018026 | 0.793459927 | 1.637941696 | 0.478491145 |
| TWIST2 | 1.040607358 | 0.924284246 | 1.171569977 | 0.510451216 |
| YTHDF1 | 1.071403293 | 0.809018787 | 1.418885488 | 0.630356507 |
| YTHDC1 | 0.915458427 | 0.601678074 | 1.392877965 | 0.679978195 |
| IGF2BP3 | 0.979746765 | 0.86459069 | 1.110240644 | 0.748416901 |
| ZNF281 | 1.054353194 | 0.758081572 | 1.466412982 | 0.753175809 |
| IGF2BP2 | 1.012372107 | 0.922885666 | 1.110535488 | 0.794546112 |
| ZNF143 | 1.067913672 | 0.630608604 | 1.808474547 | 0.806863826 |
| METTL16 | 0.965701776 | 0.706570668 | 1.319867866 | 0.826698449 |
| VIM | 1.010609918 | 0.886206959 | 1.152476174 | 0.874871785 |
| CDH1 | 0.987439445 | 0.843227405 | 1.156315191 | 0.875308609 |
| RBM15 | 1.024248479 | 0.654255659 | 1.603478597 | 0.91655927 |
| MUC1 | 0.996044982 | 0.905934403 | 1.095118591 | 0.934719094 |
| YTHDF2 | 0.987360834 | 0.714271493 | 1.364861155 | 0.938624241 |
| RBM15B | 1.008062186 | 0.745683567 | 1.362762191 | 0.958367264 |
| PARP1 | 1.003442131 | 0.804161817 | 1.252106341 | 0.975731536 |

| **Table S3：The weight factor of prognosis-related regulators** | |
| --- | --- |
| **Gene** | **Weight** |
| KIAA1429 | 0.223188583 |
| WTAP | 0.196088665 |
| SNAI1 | 0.099627323 |
| AXL | 0.055426256 |
| IGF2BP1 | 0.128025776 |
| ELAVL1 | -0.121822907 |
| CDH2 | -0.017291122 |
| ALKBH5 | -0.060846255 |
| CBLL1 | 0.131038297 |
| NANOG | -1.133543897 |

| **Table S4： Correlation analysis of core prognosis-related immological signatures** | | | | | |
| --- | --- | --- | --- | --- | --- |
| **Regulators** | **Mean±SD** | **Spearman** | | **Pearson** | |
|  |  | **r** | ***P*** | **r** | ***P*** |
| CDH2 | 3±1.688 | 0.506 | 1.11e-31 | 0.478 | 4.56e-28 |
| ALKBH5 | 4.783±0.472 |  |  |  |  |
| CDH2 | 3±1.688 | -0.439 | 1e-23 | -0.442 | 2.05e-23 |
| WTAP | 4.066±0.629 |  |  |  |  |
| CDH2 | 3±1.688 | 0.301 | 3.3e-11 | 0.292 | 1.16e-10 |
| ELAVL1 | 3.974±0.39 |  |  |  |  |
